# Comparative Transcriptome Analysis of Anthurium “Albama” and Its Anthocyanin-Loss Mutant

**DOI:** 10.1371/journal.pone.0119027

**Published:** 2015-03-17

**Authors:** Zhiying Li, Jiabin Wang, Xuequan Zhang, Li Xu

**Affiliations:** 1 Ministry of Agriculture Key Laboratory of Crop Gene Resources and Germplasm Enhancement in Southern China, Institute of Tropical Crop Genetic Resources, Chinese Academy of Tropical Agricultural Sciences, Danzhou 571737, Hainan, China; 2 Hainan University, Haikou, Hainan Province 571700, PR China; Chinese Academy of Sciences, CHINA

## Abstract

*Anthurium* is one of the most important tropical ornamental plants in the world. The traded value of *anthurium* is second only to that of tropical orchids among the tropical flowers. The spathe is the main ornamental organ and its color variation mainly arises from anthocyanin contents. Understanding the molecular regulation of spathe color will accelerate new variety creation of *anthurium*. To announce gene expression differences between *Anthurium andraeanum* ‘Albama’ and its one unique anthocyanin-loss mutant, we collected spathes of the wild-type and the mutant from two stages in spathe development (the flower separates protrude from the sheath and the spathe is fully expanded) and extracted total RNAs for transcriptome profiling. Using short read sequencing technology (Illumina), 51,955,564, 53,822,224, 54,221,990 and 52,276,418 sequencing raw reads, respectively, for wild-type and mutant in the two stages were assembled *de novo* into 111,268 unique sequences (unigenes) with a mean length of 652 bp. 47,563 unigenes had significant hits to the sequences in the Nr database, and 32,768 unigenes showed significant similarity to known proteins in the Swiss-Prot database. 28,350 and 19,293 unigenes had significant similarity to existing sequences in the KEGG and COG databases, respectively. Further, analysis of differentially expressed genes in the comparison between wild-type and mutant and between the two different developmental stages was carried out, indicating that the expression of an extensive set of genes changed as the result of mutation. Taken together, these data demonstrated that the Illumina sequencing allowed *de novo* transcriptome assembly and could obtain differentially expressed genes between *A*. *andraeanum* wild-type and the anthocyanin-loss mutant. The expression differences of *AN2* and *UFGT* might cause the anthocyanin-loss mutation.

## Introduction

*A*. *andraeanum* is a kind of monocotyledonous diploid ornamental plants that originated in Columbia and belongs to the *Araceae* family. It becomes increasingly popular in the international market and ranks second only to tropical orchid. It differs markedly from many other ornamentals in that the commercial flower consists of the brilliantly colored heart-shaped bract, called a spathe, and a cylindrical protruding inflorescence called the spadix, on which the microscopic true flowers are borne. The development of *A*. *andraeanum* flower can be divided into six stages: 1) flower not ye emerged; 2) flower is first visible; 3) flower separates protrudes from sheath separating from the base of leaf; 4) flower peduncle enlongates; 5) the spathe is half unfolded; 6) the spathe is fully expended [1].

In commercial *A*. *andraeanum* lines, the common colors of the spathe are red/pink, orange/coral and white. A few cases of green and even brown-colored spathes are also known [2]. In traditional hybrid breeding, inheritance of major spathe colors in *A*. *andraeanum* is determined by three major genes, including R, O, and M genes, which are all regulatory genes [3]. An unknown regulatory gene simultaneously suppressed the transcript levels of the structural genes *CHS*, *F3H*, and *ANS* in the white anthurium cultivar, ‘Acropolis’ [1]. The study suggested that the whites were regulatory mutants rather than structural mutants. A mutant of “Alabama” with significant different phenotype contrast to the wild-type was found: mutant plants have white spathes and pure green leaves, petioles, floral shoots, stipules and roots, but wild-type have red spathes and more red young leaves, petioles, floral shoots, stipules and young roots. The unique mutant has no red color in the whole plant and manifested as anthocyanin-loss. It is different from any other commercial anthurium species.

The phenylpropanoid pathway produces a large range of compounds derived from phenylalanine, including lignins, lignans, stilbenes and flavonoid [4, 5]. Anthocyanins are produced by a specific branch of the flavonoid pathway. Several critical structural genes in the pathway, including chalcone synthase (*CHS*), chalcone isomerase (*CHI*), flavanone 3-hydroxylase (*F3H*), flavonoid 3′-hydroxylase (*F3′H*), flavonoid 3′,5′-hydroxylase (*F3′5′H*) and dihydroflavonol 4-reductase (*DFR*) had been indentified in many plants [6]. In *A*. *andraeanum*, the major color pigments in the spathe are anthocyanins, in particular pelargonidin and cyanidin derivatives, which are accompanied by the colorless flavone C-glycosides [7].

After anthocyanin biosynthesis, UDP glucose flavonoid 3-O-glucosyltransferase (*UFGT*) plays a critical role in red color formation of flowers. Chen et al. [8] found that the *PeUFGT3* suppressed *Phalaenopsis* exhibited various levels of flower color fading that was well correlated with the extent of reduced level of *PeUFGT3* transcriptional activity. Furthermore, the transport and accumulation of anthocyanins affect the color phenotype of plant. The transport of anthocyanin to vacuolar requires the action of a glutathione S-transferase (*GST*) represented by *BZ2* in *maize* and *AN9* in *petunia* [9]. *Maize MRP3* (multidrug resistance-associated protein) would recognize the GST-cyanidin 3-O-glucoside (*C3G*) complex and pump *C3G* into the vacuole [10]. Castellarin and Di Gaspero [11] revealed that transcripts of *UFGT* and *GST* genes were absent in the green-skinned cultivar ‘Tocai friulano’, and were at least 10-fold less abundant in pale red cultivars, such as ‘Pinot gris’ and ’Gewürztraminer’, compared to fully colored cultivars.

Post-transcriptional regulation also affects anthocyanin biosynthesis. Procissi et al. [10] suggested a multilevel regulation of the *Sn* transcription factor, a member of this family, acting not only at the transcriptional but also at the post-transcriptional level [12]. Meanwhile, post-transcriptional regulation of bHLH probably controlled by *WD40* [13]. The glutathione S-transferase encoded by *Bronze2* performs the last genetically defined step in *maize* anthocyanin biosynthesis, being required for pigment sequestration into vacuoles. Pairoba and Walbot [14] found the accumulation of *Bronze2* appeared to be limited by stringent post-transcriptional regulation.

Anthocyanin production is differently regulated in monocot and dicot species. In the monocot *maize*, the anthocyanin biosynthesis genes are activated as a single unit by a ternary complex of *MYB-bHLH-WD40* transcription factors (MBW complex). In the dicot Arabidopsis, anthocyanin biosynthesis genes can be divided in two subgroups: early biosynthesis genes (EBGs) are activated by co-activator independent *R2R3-MYB* transcription factors, whereas late biosynthesis genes (LBGs) require an MBW complex. In addition, a complex regulatory network of positive and negative feedback mechanisms controlling anthocyanins synthesis in Arabidopsis had been described [15].

Anthocyanin biosynthesis, glycosylation, transport and accumulation all influence flower color. All the data indicate that, although transcription factors from different species are involved in the same biosynthetic process, they are characterized by a different specificity in their target genes. Bioinformatic analysis may therefore help in selecting the proper heterologous regulators. In this study, we built comparative transcriptomes between anthurium wild-type sample of red spathe and mutant sample of white spathe both in stage 6 and stage 3. As a result, we identified the genes that related to anthocyanin biosynthesis, glycosylation, transport and accumulation from all differential expression genes sequences. At last, we concluded that *UFGT*, *GST* and *MRP* genes expressing in lower level might cause the mutation of anthocyanin-loss, although a lot of genes expressing level had changed.

## Result

### Illumina sequencing and *de novo* assembly

**Fig 1:**
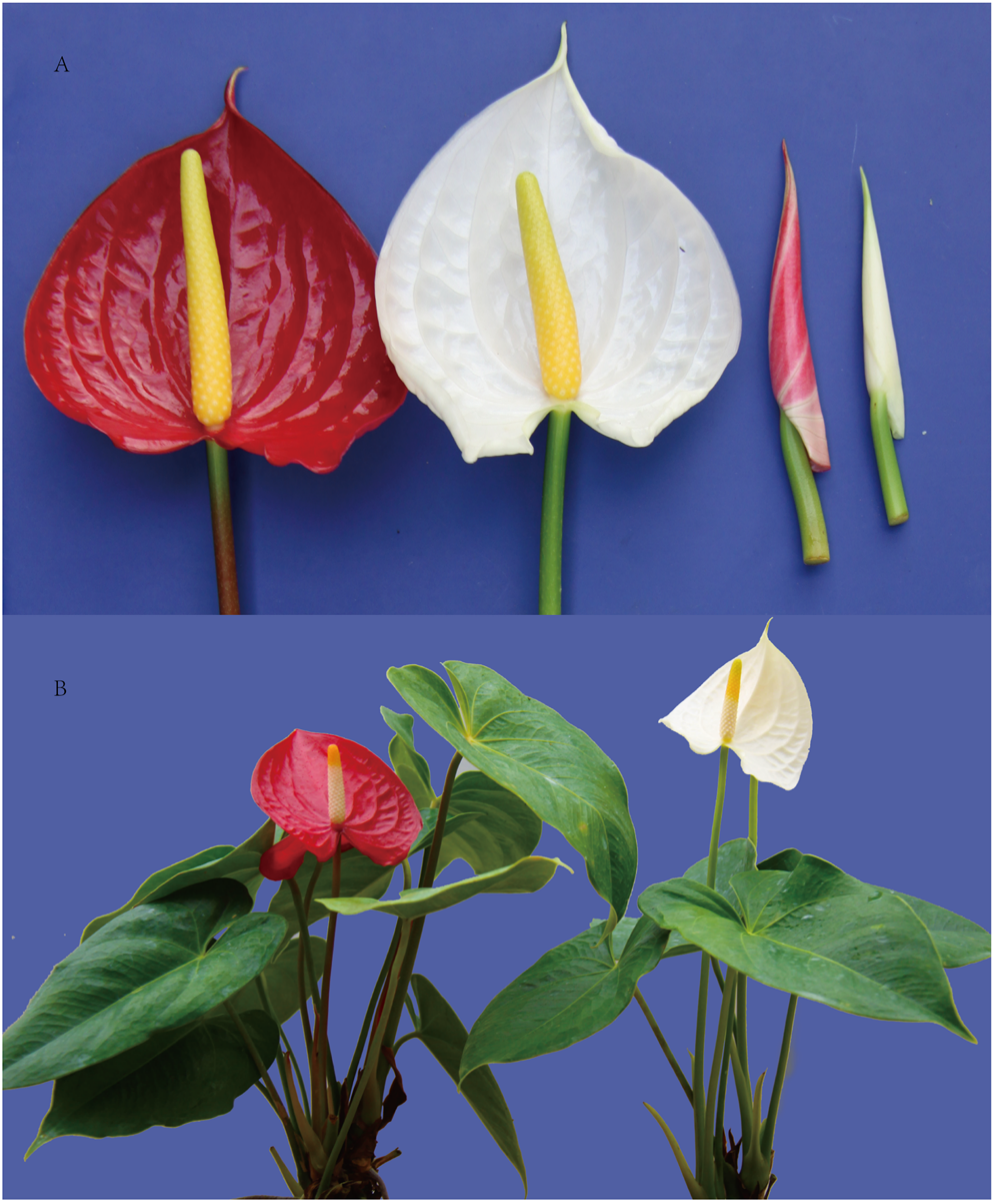
Figure of *A*. *andraeanum*. (A) Spathe of *A*. *andraeanum* and its anthocyanin-loss mutant in flower developmental stage 3 and stage 6. (B) *A*. *andraeanum* and its anthocyanin-loss mutant in flower developmental stage 6.

**Fig 2:**
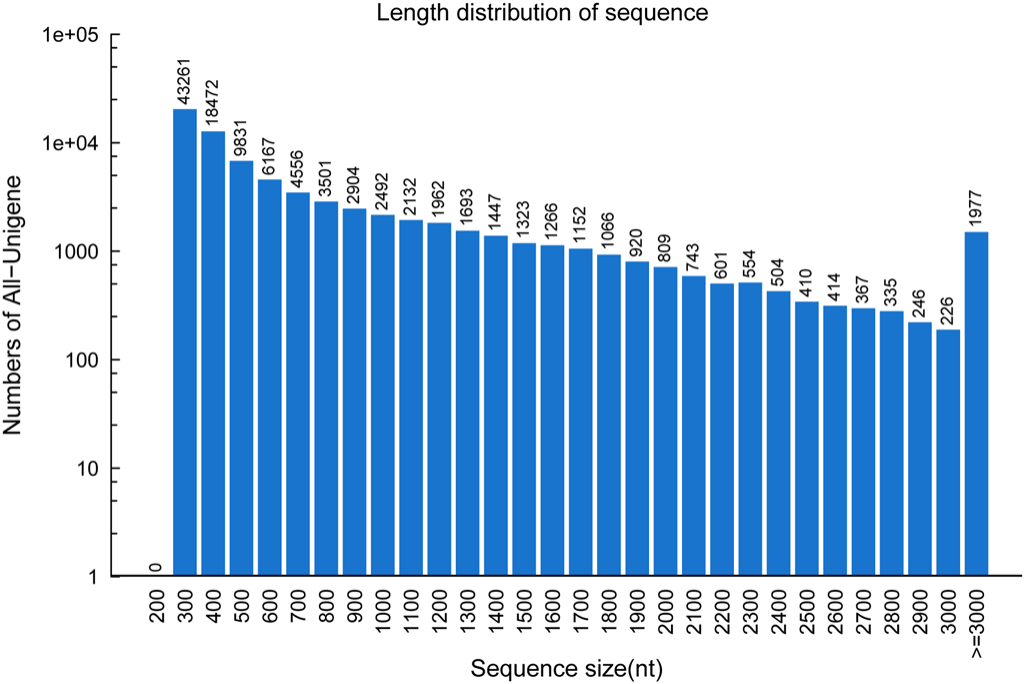
The length distribution of assembled sequences. The reads from four samples were assembled into 111,268 transcripts.

In this study, four cDNA samples from the spathes of wild-type *A*. *andraeanum* “Alabama” in stage 6 (WS6) and stage 3 (WS3) and its anthocyanin-loss mutant in stage 6 (MS6) and stage 3 (WS3) were prepared and subjected to Illumina deep sequencing (Fig. 1). The output of sequenced data from WS6, WS3, MS6 and MS3 were 51,955,564, 53,822,224, 54,221,990 and 52,276,418 qualified Illumina reads respectively with 90 bp mean length. Then, using trinity [16], these clean reads were assembled to unigene sequences. Finally, unigenes of the four samples were summarized into an All-unigene with 111,268 sequences with mean size of 652 bp, which including all non-redundant unigene sequences of both four samples (Table 1). Fig. 2 showed the distribution of transcripts length, with the length of transcripts ranges from 200 to 11534.

**Table 1:** Statistics of Illumina transcriptome sequencing of the *anthurium*.

	*WS3*	*MS3*	*WS6*	*MS6*
**Reads(n)**	54,221,990	52,276,418	51,955,564	53,822,224
**Base number(bp)**	4,879,979,100	4,704,877,620	4,676,000,760	4,844,000,160
**Total residues(bp)**	72584422			
**Number of transcripts(n)**	111268			
**Average length(bp)**	652			

### Annotations of sequences

**Fig 3:**
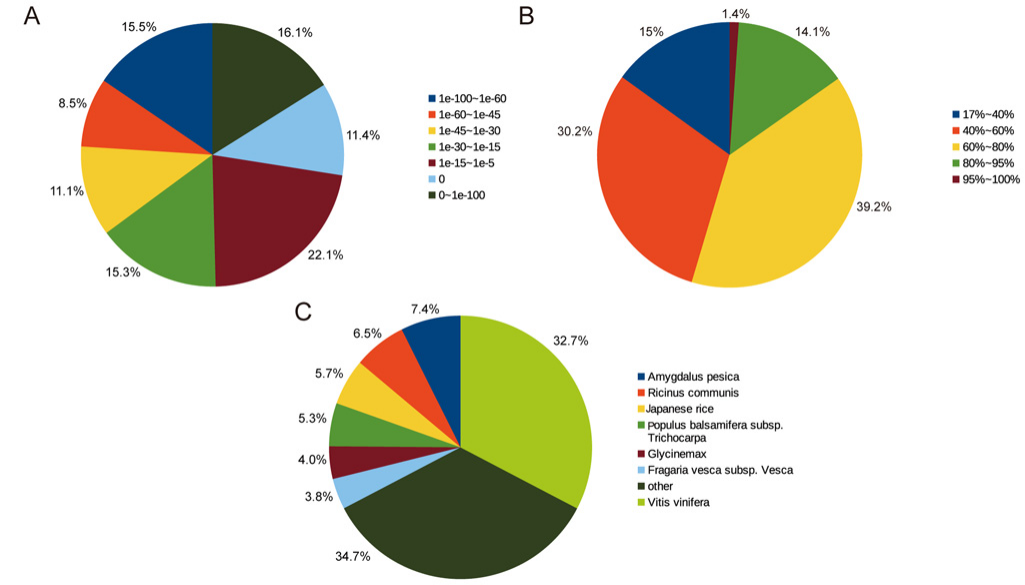
Summary for results of BLASTx against NCBI NR database. (A) E-value distribution of BlastX results. (B) Similarity distribution of Blastx results. (c) Species distributions of BlastX rsults.

For annotation, unigene sequences of *A*. *andraeanum* were first searched using BlastX against the non-redundant (Nr) database of NCBI with a cut-off E-value of 1e-5. Using this approach, 47,563 unigenes (43.4% of all unigene sequences) returned an above cut-off BlastX result. The E-vaule distribution of BlastX result was shown in Fig. 3A. Of the search results, 11.4% of the matches were with a E-value of 0, meanwhile, 31.69% of the matches were with a E-value less than 1e-60. Correspondingly, the similarity ditribution of best matches were shown in Fig. 3B, 15.4% of the matches were of high similarity ranging from 85% to 100% and 39.2% of the hits were of similarity ranging from 60% to 80%. Moreover, the species-based distribution of best matches were shown in the Fig. 3C. The result of homology analysis indicated that 32.7% of the sequences of *A*. *andraeanum* showed the greatest similarity to proteins of *Vitis vinifera*, whilst proteins of *Amygdalus pesica* (7.4%), *Ricinus communis* (6.5%), *Populus balsamifera subsp*. *tricholarpa* (5.3%), *Clycine max* (4.0%) and *Fragaria vesca subsp*. *Vesca* (3.8%) showed a lower similarity to sequences of *A*. *andraeanum*. Then, these unigene sequences were second searched using BLASTx against the Swiss-Prot database using a cut-off E-value of 1e-5, with 32,768 unigenes (29.8% of all unigene sequences) returned an above cut-off BLAST result.

**Fig 4:**
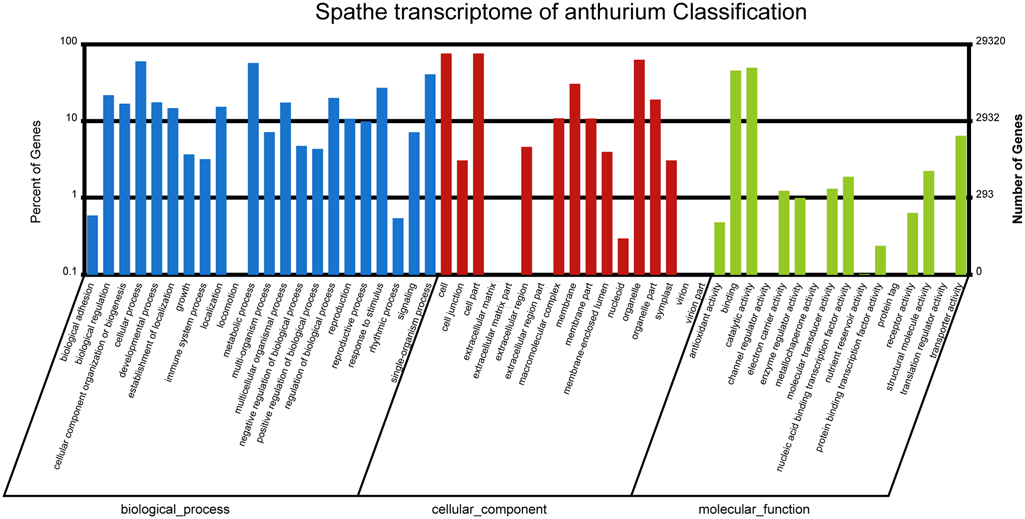
GO assignments for transcriptome of *A*. *andraeanum*.

GO assignments were used to classify the functions of the unigenes based on Nr annotation using blast2go [17]. Of the unigenes with significant hits in Nr database, 28,289 unigenes were categorized into 64 functional groups (S1 Table). Amongst the sub-categories of three main GO categories, cell (10.6%), cell part (10.6%), organelle (8.6%), cellular process (7.9%), metabolic process (7.7%), catalytic activity (7.0%) and binding (7.0%) occupied the major proportion. Rather, only a few unigenes were assigned into categories of virion, viron part, extracellular matrix part, metallochaperone activity, channel regulator activity, protein tag and viral reproduction (Fig. 4).

**Fig 5:**
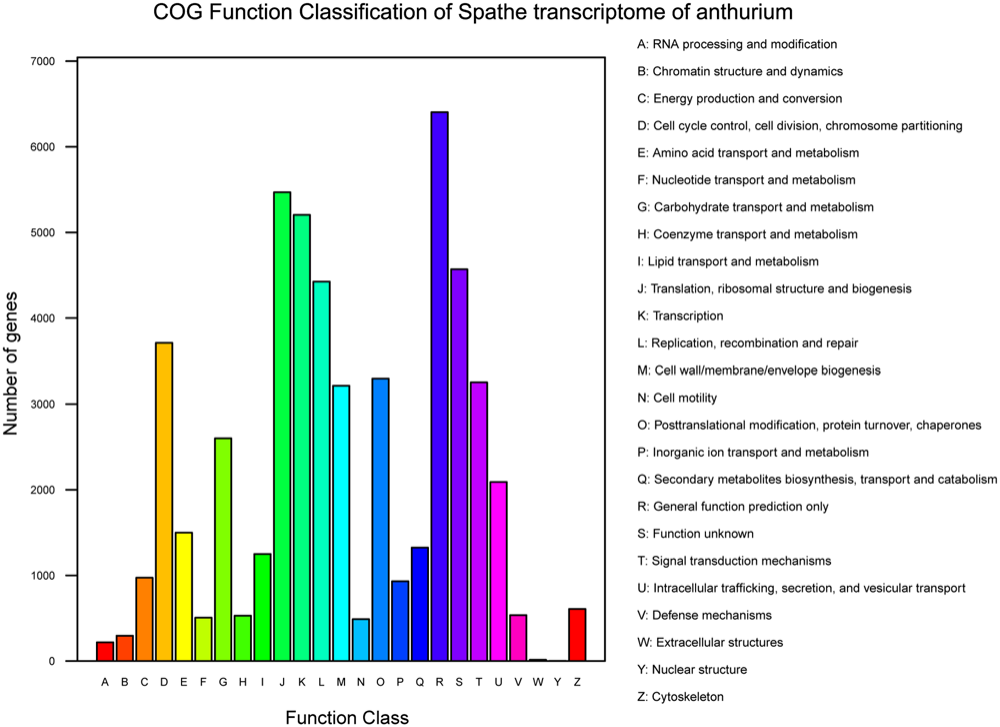
COG Functional classification of transcriptome of *A*. *andraeanum*.

To further evaluate the completeness of our transcriptome library and the effectiveness of our annotation process, we searched the annotated sequences for the genes involved in COG classifications. In total, out of 47,563 Nr hits, 19,293 sequences have a COG classification (Fig. 5). These sequences were classfied into 24 categories, of which the categories included General function prediction only (11.7%), Translation, ribosomal structure and biogenesis (11.2%), Transcription (9.5%), Replication, recombination and repair (8.4%) and Function unknown (8.3%) were the top 5 categories that sequnces be categorized (S2 Table). In the meanwhile, of the 24 categories, Defense mechanisms (13; 0.023%) and Nuclear structure (2; 0.0036%) were the least represented.

To identify the biological pathways that are active in *A*. *andraeanum*, we mapped the 47,563 annotated sequences to the reference canonical pathways in Kyoto Encyclopedia of Genes and Genomes (KEGG) [18]. In total, we assigned 28,350 sequences to 129 KEGG pathways (S3 Table). Amongst the kegg pathways, Metabolic pathways (16.5%), Spliceosome (7.8%), Biosynthesis of secondary metabolites (5.4%), Endocytosis (4.4%), RNA transport (4.3%), Ether lipid metabolism (3.9%), Plant-pathogen interaction (3.6%) and mRNA surveillance pathway (3.2%) were the highly represented pathways. Specially, ABC transporters (0.52%), Phenylpropanoid biosynthesis (0.68%), Flavonoid biosynthesis (0.43%), Glutathione metabolism (0.32%), Phenylalanine metabolism (0.32%), Flavone and flavonol biosynthesis (0.19%) and Anthocyanin biosynthesis (0.02%) that were closely associated with anthocyanin metabolism were represented.

Furthermore, *A*. *andraeanum* unigene sequences were aligned against several protein databases using BlastX (Evalue<1e-5) untill as much as unigene sequences have hits. CDS of unigenes have no hit in blast were predicted by ESTScan [19] and then translated into peptide sequences. In the end, we obtained a “Blast-CDS” data including 47,123 unigene sequences and an “ESTscan-CDS” including 5,243 EST sequences.

### Unigene expression analysis

**Fig 6:**
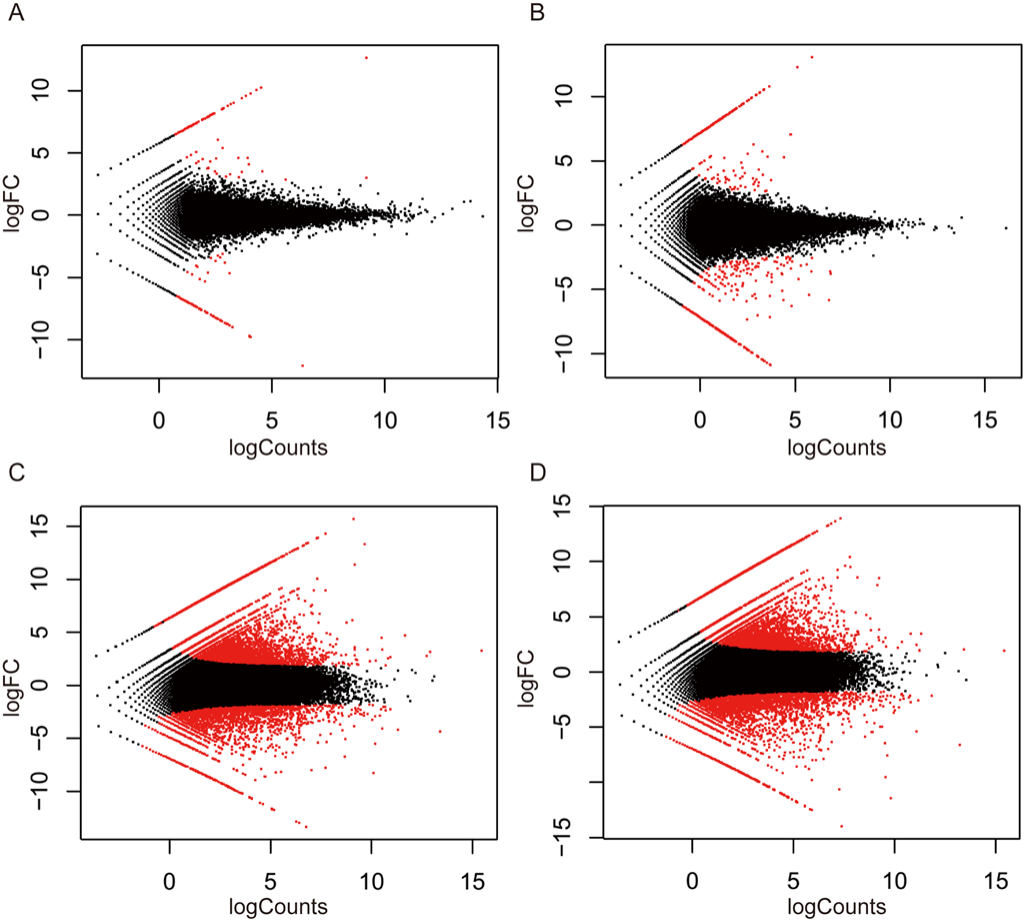
Log-fold changes in gene expression. (A) Log-fold changes in gene expression in WS6 vs MS6. (B) Log-fold changes in gene expression in WS3 vs MS3. (C) Log-fold changes in gene expression in MS6 vs MS3. (D) Log-fold changes in gene expression in WS6 vs WS3.

**Fig 7:**
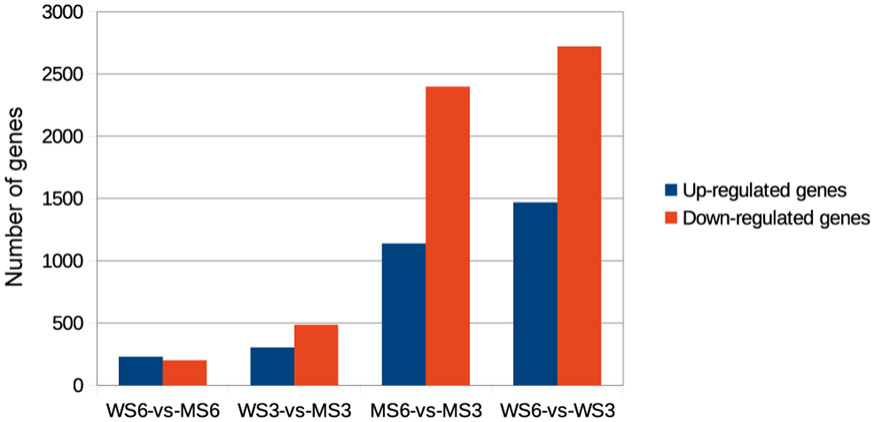
Differently expressed genes in WS6 vs MS6, WS3 vs MS3, MS6 vs MS3, WS6 vs MS3. WS6 vs MS6 refers to the comparison between expanded spathe in wild-type and mutant. WS3 vs MS3 refers to the comparison between unexpanded spathe in wild-type and mutant. MS6 vs MS3 refers to the comparison between unexpanded spathe and expanded spathe in mutant. WS6 vs WS3 refers to the comparison between unexpanded spathe and expanded spathe in wild-type. “P-value<0.001, the absolute value of Log2 fold change (Log2 FC)≥2 and FPKM≥1” were used as the threshold to determine the significance of gene expression differences.

Genome-wide expression analysis was carried out to study the differences between spathes of wild-type and mutant during different developmental stages. The analysis found that 428, 787, 3,534 and 4,187 genes had different expression levels in the comparisons between WS6 and MS6 (WS6 vs MS6), between WS3 and MS3 (WS3 vs MS3), between MS6 and MS3 (MS6 vs MS3) and betweem WS6 and WS3 (WS6 and WS3) respectively (P-value <0.001; Log2 fold changes≥2 or ≤-2). Fig. 6A, 6B, 6C and 6D showed the expression pattern for WS6 vs MS6, WS3 vs MS3, MS6 vs MS3 and WS6 vs MS3 respectively. Correspondingly, the Fig. 7 illustrated the gene expression changes: for WS6 vs MS6, there are 199 up-regulated genes and 229 down-regulated genes; for WS3 vs MS3, there were 484 up-regulated genes and 303 down-regulated genes; for MS6 vs MS3, there were 1137 up-regulated genes and 2397 down-regulated genes; for WS6 vs WS3, there were 1467 up-regulated genes and 2720 down-regulated genes.

Using GO-TermFinder [20], GO functional enrichment analysis was carried out to characterize the functions of differentially expressed genes (DEGs). The result revealed that DEGs were enriched into 30 functional groups (Corrected Pvalue<0.05) (S4 Table). DEGs in WS6 vs MS6 were enriched in the categories of virion part and virion and DEGs in WS3 vs MS3 were enriched in the categories of membrane, metabolic process, catalytic activity and cellular component organization, showing a different functional view of DEGs between wild-type and mutant in spathe developmental stage 6 and stage 3. Furthermore, the DEGs in WS6 vs WS3 and MS6 vs MS3 were both enriched in 14 categories, inlcuding symplast, biological regulation, growth, pigmentation, envelope etc. (S4 Table), indicating that both wild-type and muntant have similar and extensive changes in gene expression level during different developmental stages of spathe.

To further explore the functions of DEGs, KOBAS [21] was used for pahtway enrichment annalysis. The DEGs in MS6 vs MS3 and WS3 vs MS3 were both enriched in the Homologous recombination pathway, while the DEGs in MS6 vs WS6 and WS6 vs WS3 were both enriched in the pathways of the Ubiquinone and other terpenoid-quinone biosynthesis, RNA transport, mRNA surveillance pathway, Plant hormone signal transduction, Oxidative phosphorylation, Flavonoid biosynthesis, Regulation of autophagy etc. (Table 2; the complete information were summaried in S5 Table). In this study, we focused on genes involved in anthocyanin biosynthesis and pathways related to anthocyanin biosynthesis such as ABC transporters, Glutathione metabolism, although changes of anthocyanin biosynthesis affected expression of many other pathways.

**Table 2:** List of enriched pathways of differently expressed genes.

*Pathway*	*Pathway.ID*	*Bn*	*Nt1*	*Nt2*	*Nt3*	*Nt4*
**Purine metabolism**	ko00230	448	10	3	42	33
**RNA polymerase**	ko03020	555	1	4	20	27
**Homologous recombination**	ko03440	1927	8	14	256	239
**Phosphatidylinositol signaling system**	ko04070	539	1	4	58	64
**Glyoxylate and dicarboxylate metabolism**	ko00630	214	9	5	12	10
**Thiamine metabolism**	ko00730	12	2	1	0	1
**Ribosome biogenesis in eukaryotes**	ko03008	663	7	3	20	29
**Cutin, suberine and wax biosynthesis**	ko00073	63	1	7	22	9
**Endocytosis**	ko04144	3461	23	32	118	156
**Biosynthesis of secondary metabolites**	ko01110	1624	12	8	96	118
**Aminoacyl-tRNA biosynthesis**	ko00970	240	5	10	21	22
**Ubiquinone and other terpenoid-quinone biosynthesis**	ko00130	1771	8	14	250	232
**RNA transport**	ko03013	1955	11	25	211	232
**mRNA surveillance pathway**	ko03015	1467	6	19	178	183
**Plant hormone signal transduction**	ko04075	819	12	20	112	106
**Metabolic pathways**	ko01100	7383	72	112	729	690
**Glycerophospholipid metabolism**	ko00564	2426	29	53	231	235
**Starch and sucrose metabolism**	ko00500	866	5	11	94	94
**Protein processing in endoplasmic reticulum**	ko04141	660	6	6	30	35
**Pentose and glucuronate interconversions**	ko00040	2009	9	20	255	251
**Oxidative phosphorylation**	ko00190	304	2	8	51	45
**Peroxisome**	ko04146	191	2	4	5	9
**Fructose and mannose metabolism**	ko00051	121	2	1	3	1
**Other glycan degradation**	ko00511	192	3	5	29	30
**Flavonoid biosynthesis**	ko00941	162	1	0	36	36
**Regulation of autophagy**	ko04140	120	1	0	3	2
**DNA replication**	ko03030	144	1	3	27	20
**Phenylalanine metabolism**	ko00360	161	3	7	22	22
**Nitrogen metabolism**	ko00910	97	1	0	1	6
**Fatty acid biosynthesis**	ko00061	77	1	1	1	3
**SNARE interactions in vesicular transport**	ko04130	159	3	5	30	25
**Sulfur metabolism**	ko00920	132	0	1	3	6
**Biosynthesis of unsaturated fatty acids**	ko01040	138	4	1	25	20
**ABC transporters**	ko02010	245	4	7	12	9
**Glutathione metabolism**	ko00480	149	2	1	18	22
**Propanoate metabolism**	ko00640	95	0	1	4	2

Bn (background number), the total number of transcripts be annotated into the certain pathways. Nt1, the number of differently expressed genes in WS6 vs MS6. Nt2, the number of differently expressed genes in WS3 vs MS3. Nt3, the number of differently expressed genes in MS6 vs MS3. Nt4, the number of differently expressed genes in WS6 vs WS3.

### Detection of sequences related to anthocyanin biosynthesis

The plant flavonoid pathway lead to flavones and anthocyanins synthesis [2]. According to the flavonoid pathway and all differentially expressed sequences data, we screened 18 fragments which are homologous to the genes related to phenylpropanoid pathyway, flavonoid biosynthesis pathway and anthocyanins transport pathway (Table 3; the complete information of the 18 DEGs was summarized in S6 Table).

**Table 3:** Differentially expressed genes related with anthocyanin.

			*WS6-vs-MS6*	*WS3-vs-MS3*	*MS6-vs-MS3*	*WS6-vs-WS3*
GeneID	Homologous Genes	Homologous Genes ID	log2 FC	log2 FC	log2 FC	log2 FC
c50000092719_g1_i1	*Solanum lycopersicum* anthocyanin 2 (AN2)-like mRNA	gb|FJ744760.1	-6.6038		-7.3869	
c50000028404_g1_i1	PREDICTED: myc anthocyanin regulatory protein [*Vitis vinifera*]	gb|ABM92332.3	1.0146	-0.0804	2.4721	1.3581
c3000008272_g1_i1	*Anthurium andraeanum* flavanone 3-hydroxylase (F3H)	gb|DQ972935.1	0.1115	0.5222	1.9660	2.3576
c30000038819_g1_i1	Anthocyanidin 3-O-glucosyltransferase [*Iris hollandica*]	gb|BAD83701.1	7.7364			-8.5307
c30000017694_g1_i1	Flavonoid 3′-hydroxylase [*Vitis vinifera*]	gb|BAE47004.1	0.5884	0.3344	3.0783	2.8053
c2000008556_g1_i1	Putative leucoanthocyanidin reductase [*Diospyros kaki*]	gb|BAH89267.1		0.4518	9.3634	9.8041
c2000007630_g2_i1	Dihydroflavonol 4-reductase [*Anthurium andraeanum*]	gb|ABC94578.1	0.2640	0.1768	2.4234	2.3174
c2000005858_g4_i1	putative flavonoid/anthocyanin regulator [*Anthurium andraeanum*]	gb|AAO92352.1	2.2553	0.7610	2.9307	1.4103
c200000509_g5_i1	Anthocyanidin 3-O-glucosyltransferase [*Manihot esculenta*]	gb|CAA54614.1	9.7867	3.8887	5.9979	-0.7137
c2000004936_g2_i1	chalcone synthase [*Anthurium andraeanum*]	gb|ABE01413.1	0.4842	-0.1429	3.0880	2.4421
c2000003002_g2_i1	*Zea mays* PL transcription factor	gb|AY135019.1	5.0999	0.2855	10.8134	5.2088
c2000003002_g1_i1	Anthocyanin regulatory C1 protein [*Zea mays*]	gb|AAK09327.1	0.7429	-0.5487	4.7550	3.4361
c2000002414_g4_i1	anthocyanidin reductase [*Malus x domestica*]	gb|AAZ79363.1	-0.0224	0.2887	4.3490	4.6414
c5000006781_g1_i1	*Phalaenopsis schilleriana* anthocyanin related UMyb7 mRNA	gb|FJ039861.1	1.1509	0.5660	1.9873	1.3768
c3000006903_g1_i1	*Camellia sinensis* chalcone isomerase	gb|AHB32112.1	-1.1354	-1.5126	-2.0135	-2.4067
c2000001891_g4_i1	glutathione S-transferase [*Musa acuminata AAA Group*]	gb|AGH14251.1	-0.4508	-0.9760	-2.0758	-2.6192
c2000001570_g2_i1	*Fragaria vesca subsp*. *vesca* ABC transporter C family member 3-like	ref|XP_004309817.1	-4.0358	-1.9712	0.7593	3.5919
c2000004629_g2_i1	anthocyanidin 5,3-O-glucosyltransferase [*Anthurium andraeanum*]	gb|AGS12592.1	0.1269	-0.4704	1.8300	1.2139

The analysis of differential gene expression in MS6 vs WS6 showed that c3000005417_g1_i11 (*DFR*), c30000038819_g1_i1 (*UFGT*) and c200000509_g5_i1 (*UFGT*) were down-regulated in MS6. And c200000509_g5_i1 was also down-regulated in differential gene expression analysis of MS3 vs WS3. Especially, c50000092719_g1_i1 (AN2), homologous AN2 gene, only expressed in the full spread spathe of the mutant (MS6) but very low in the unexpanded spathe of the mutant (MS3). We speculated that the expressional difference of AN2 between the wild type and the mutant and between different developmental stages of spathe affected the biosynthesis of anthocyanin, although AN2 were lowly expressed MS6 with FPKM value of 9.39 (25 fragments count). And other genes’ expression levels may also be associated with the color mutation. We isolated the full-length cDNA of *AN2* from *A*. *andraeanum* and designated as *AnAN2*. Then the function of *AnAN2* were confrimed by a inhibition of anthocynains biosynthesis phenotypes in *A*. *thaliana* caused by ectopic expression of *AnAN2*. *AnAN2* driven by the CaMV 35S promoter was transformed into *A*. *thaliana* ecotype ‘Columbia’ plants. Independent *35S*::*AnAN2* transgenic *A*. *thaliana* plants were screened on MS medium containing 50 mg/L Hyg. The *35S*::*AnAN2* transgenic plants appeared significantly anthocyanin biosynthesis phenotypes both in seedlings and adult plants, indicating *AnAN2* act as a negative regulator of anthocyanin biosynthetic pathway (S1 Fig.).

The Analysis of differrential gene expression in MS6 vs MS3 and WS6 vs WS3 showed that c50000028404_g1_i1 (*MYCA1*), c30000017694_g1_i1 (*F3’H*), c2000008556_g1_i1 (*LAR*), c2000004936_g2_i1 (*CHS*), c200000509_g5_i1 (*UFGT*), c2000003002_g2_i1 (*PL*), c2000003002_g1_i1 (*C1*), c2000002414_g4_i1 (*ANR*) and c2000004629_g2_i1 (anthocyanidin 5,3-O-glucosyltransferase) were both up-regulated in MS3 and WS3, while c3000006903_g1_i1 (*CHI*) and c2000001891_g4_i1 (*GST*) were down-regulated in MS3 and WS3. *MYCA1* was reported in *vitis vinifera*, which may regulated *ANR* and *UFGT* and response for anthocyanin accumulation [22]. *Zea Mays PL* transcription factor (*PL*) and *C1* were belong to *C1*/*Pl* gene family, of which *C1* controls pigmention of the kernel and *Pl* controls pigmention of vegetative and floral organs [23]. This result indicated that the expresion of many structural genes and regulators associated with anthocyanin synthesis reduced in expanded spathe compared to unexpanded spathe.

### Verification of comparative transciptome results

**Fig 8:**
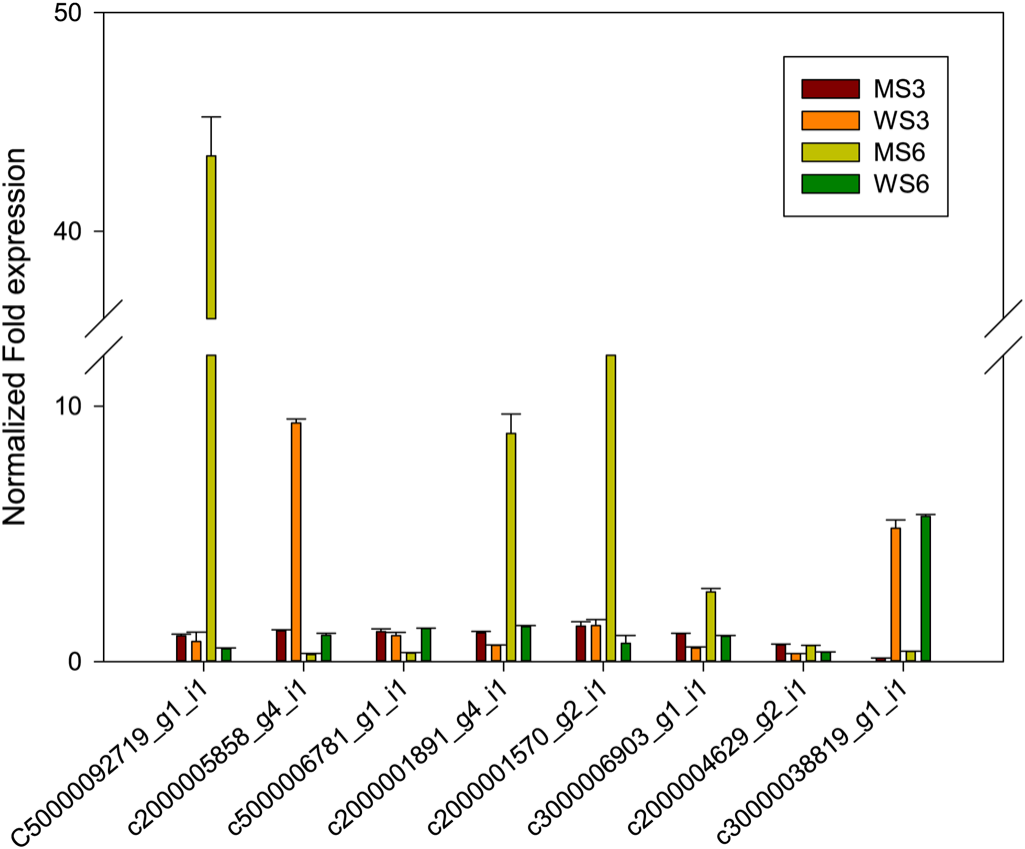
Real-time qPCR validation of genes related to anthocyanin. Data were normalize against a reference of *A*. *andraeanum* actin gene(gi|397881472). Real-time PCR reactions were set up with three biological replications and three technical replicates per experiment. Mean values and standard errors (bars) were obtained from independent experiments. The error bars indicate SD.

Several genes whose expression was altered to varying degrees in the mutant were chosen for verification of the comparative transciptome results. The results of qRT-PCR perfomed on RNA prepared from the conserved full spread young spathe and unexpanded at-80℃ were agreement with the alterations in gene expression detected by the transcriptome analysis. This agreement was seen for the direction of change, and was also generally seen for the magnitude of change, in gene expression (Fig. 8). For these experiments, cDNA aliquots were taken from the same samples used for transcriptome sequencing. These results indicated that transcriptome sequencing accurately reflected genome-wide changes between the wild type and the mutant. For the low expression of *AN2* in our transcriptome data, we confirmed especially the qPCR products was specific to *AN2* by gel electrophoresis and sequencing (S2 Fig.).

### Anthocyanin accumulation

To examine the accumulation of anthocyanin in the *A*. *andraeanum*, spathe, petiole and leaf extracts were subjected to high-performance liquid chromatography (HPLC) analysis. The HPLC data show that the main anthocyanins, including peonidin-rutinoside, anthocyanidin-rutinoside and pelargonidin-rutinoside, apeared in the wild-type spathe, petiole and leaf but did not in the both of mutant spathe, petiole and leaf, indicating that the mutant was anthocyanin-loss (S7 Table).

## Discussion

### Sequencing and annotation

With the devlopment of RNA-seq, transcriptome has become an available and successful alternatvie to in-depth detect difference of gene expression in wild-type and mutant or different cutivars of certain species, such as Enoch et al. [24] characterized a natrual dicromatism of the annual fish *Nothobranchius furzeri* through RNA-seq. To date, anthocyanin biosynthesis was explored by RNA-seq in many studies, such as Benhong Wu et al. [25] carried out a genome-wide transcriptional profiles of berry skins of two cultivars of *vitis vinifera* in which anthocyanin biosynthesis is sunlight-dependent and independent and Daqiu Zhao et al. [26] revealed coordinated expression of anthocyanin biosynthetic genes mediating yellow formation in *Paeonia lactiflora Pall* by transcriptome sequencing.

Anthurium is one of the most important tropical ornamental plants in the world, but the genomic information available for this species was still limited, although Danqing Tian et al. have characterzied the Anthurium transcriptome of a mixed sample of seedlings of cold treated and control plants [27]. We constructed a transcriptome of four samples from spathes of wilde-type and anthocyanin-loss mutant in flower developmental stage 6 and stage 3, which provided a more sufficent and detailed transcriptome information of spathes and will faccilitate the subsequent stuties. Interestingly, a different species-based distribution of best matches of BlastX searching again NR database compared to the transcriptome Danqing Tian et al. characterized was presented, the latter showed the closest species was *Oryza sativa* and followed by *A*. *thaliana*, while our results showed that the closest species was *Vitis vinifera* and followed by *Amygdalus pesica*, as the number of sequences annoted increased and tissue specificity of gene expression.

### Structural genes and regulators related with anthocyanin biosynthesis

Anthocyanin represents the major red, purple, violet and blue pigments in many flowers and fruits. It is produced by a specific branch of the flavonoid pathway, which is differently regulated in dicot and monocot species. In the dicot, such as Arabidopsis, anthocyanin biosynthesis genes can be divided in two subgroups: early biosynthesis genes (EBGs), i.e. *CHS*, *CHI*, *F3H*, *F3’H*, *FLS*, and the late biosynthesis genes (LBGs), i.e. *DFR*, *ANS/LDOX*, *UFGT*, *LAR*, *ANR*. A regulatory system based on the cooperation between *MYB* and *bHLH* proteins that control floral pigmentation is common in many dicotyledonous species. In *petunia* and *morning glory*, an MYB-bHLH-WD40 transcription factors (MBW complex) and a regulatory network similar to that of *Arabidopsis* has been identified. In monocot *maize* (*Zea mays*), two types of transcription factors, a MYB-related protein and a bHLH-containing protein, interact and activated the anthocyanin biosynthetic genes (*CHS*, *CHI*, *F3H*, *DFR*, *ANS/LDOX* and *UFGT*) as a single unit [15]. Although transcription factors from different species are involved in the same biosynthetic process, they are characterized by different target genes. In *maize*, mutations in the *pr1* locus lead to the accumulation of pelargonidin (red) rather than cyaniding (purple) pigments in aleurone cells where the anthocyanin biosynthetic pathway is active [28]. The mutation of anthocyanin-loss may rise from the change of the genes in anthocyanin biosynthesis, glycosylation, acyltransferation and transport.

Regulation of anthocyanin biosynthesis in spathe differs from other described species, because dihydroflavonol 4-reductase (*DFR*) is a key regulatory point and a complex mix of developmental and environmental control signals in described plants [2]. The flavonoid pathway was regulated in a spatial and temporal way during plant development. Regulation of structural genes expression is orchestrated by a ternary complex involving transcription factors from the *R2R3-MYB*, basic helix-loop-helix (*bHLH*), and *WD40* classes [29]. Repressors of *MYB*, one special kind of *bHLH* protein, could form polymer with *MYB* activator to repress transcriptional activator. They could competitive bind bHLH promoter binding domain with *MYB* activator to repress bHLH transcription factors. They could competitive bind *bHLH* transcription factor with *MYB* activator to inhibit the formation of transcriptional activation complexes. They also could competitive bind promoter binding region of structural genes to stop their transcription [30]. In this study, *DFR* had no significant difference between the wild-type samples and mutant samples. Meanwhile, *CHS*, *F3H* and *ANS* were also absent in the differential expressed genes list of comparison of wild-type and mutant. Other genes, such as *CHI* and *ANR*, were with higher level in the full spread spathe of the mutant than that of the wild-type. It meant that these structural genes and regulators were neither responsible for the anthocyanin-loss mutation, although some regulators changed.

Amongst differential expressed regulator genes, *PL*, *MYCA1*, *C1*, *MYB1* and *UMYB7* expressed with higher level in wild-type than in mutant. Interestingly, *AN2*, which positively regulates anthocyanin biosynthesis in lily [31], had 0 fragments count in wild-type full spread spathe, but 25 fragments count in that of the mutant. Using the same samples, young full spread spathe, qRT-PCR verified the result. Meanwhile, *ANR*, *C1*, *CHS*, *DFR*, *LAR*, *F3’H* and *F3H* were both up-regulated in MS3 and WS3 in the comparasion of MS6 vs MS3 and WS6 vs WS3, indicating that the genes or regulators related to anthocyanin synthesis expressed differently in different development of the spathe as reported by Collette [1]. However, *AN2*, *ABC* and *UFGT* showed a differently changing trend. This may be the result of mutation in *A*. *andraeanum*.

Glycosylation, acyltransferation and transportation play important roles in keeping anthocyanin stable and demonstrating different colors in vacuole [9]. The identified *UFGT* genes had obvious expressional difference, which may the key genes lead to anthocyanin-loss of mutant. The genes involving in anthocyanin transportion we identified are homologous to *GST* and *MRP*. They were both significiantly up-regulatd in mutant than in wild-type with changes from 2 to 4 fold respectively, suggested that anthocyanin may negatively feedback to *GST* and *MRP*. So, we hypothesized the anthocyanin-loss mutation were caused by some regulators, such as AN2 and key genes of anthocynin glycosylation *UFGT*.

## Conclusions

Summarily, this study successfully discovered the differentially expressed genes and regulators between the wild-type and the anthocyanin-loss mutant through comparison of the two transcriptome data. We hypothesized the anthocyanin-loss mutation are caused by expression changes of AN2 and UFGT genes. This hypothesis needs further verification.

## Materials and Methods

### Plant sample preparation and RNA isolation

*A*. *andraeanum* “Alabama” wild-type and its anthocyanin-loss mutant plants (New Plant Variety right: ZL201310140892.0, The Office for the Protection of New Varieties of Plant, MOA, P.R. China) were collected from a greenhouse located in the experimental area at the Institute of Tropical Crop Genetic Resources, Chinese Academy of Tropical Agricultural Sciences (CATAS). Spathe tissues in stage 3 and stage 6 of the wild-type and the mutant were physically isolated and immediately frozen in liquid nitrogen. Total RNA was extracted from the spathe without spadix dehydrated for 8 min at 65 ℃ using CTAB extraction method. The RNA samples were treated with 10 units of DNaseI (Takara) for 30 min at 37℃ to remove the genomic DNA. The quantity and quality of the isolated total RNA was examined using spectrophotometry and gel electrophoresis.

### Library preparation for transcriptome analysis and sequencing

Poly-A-containing mRNAs were purified from the total RNA samples using the OligoTex mRNA mini kit (Qiagen). The mRNA was then fragmented into small pieces using an RNA fragmentation kit (Ambion). Using these short fragments as the templates, the first cDNA strand was synthesized using random hexamer primers and reverse transcriptase (Invitrogen), and the second-strand cDNA was synthesized using DNA polymerase I and RNase H. The cDNA fragments were purified using the QiaQuick PCR extraction kit (Qiagen) and resolved with EB buffer for end reparation and poly (A) addition. The short fragments were then connected with sequencing adapters, and the products were subsequently purified and amplified via PCR to create the final cDNA libraries. The cDNA library was sequenced using Illumina HiSeq 2000, and the sequencing-derived raw image data were transformed by base calling into sequence data. The raw reads were cleaned by the trimming of adaptor sequences, empty reads and ambiguous nucleotides (‘N’ in the end of the reads). The reads obtained were then assembled using the Trinty software [16]. In the final step, BLASTX alignments (evalue<1e-5) between unigenes and protein databases, including Nr, Swiss-Prot, Kyoto Encyclopedia of Genes and Genomes (KEGG) and Cluster of orthologous group (COG), were performed, and the best alignment results were used to decide the sequence direction of the unigenes. When a unigene happened to be unaligned with none of the above databases, ESTScan software was used to predict its coding regions and to decide its sequence direction [19].

### Functional annotation and classification

The assembled unigenes were compared with the sequences in the NCBI non-redundant protein (Nr) and Swiss-Prot protein databases using the BlastX algorithm with an evalue cut-off of 1e-5. The functional annotation by gene ontology (GO) terms was performed using the BLAST2GO program [17]. After getting GO annotations, WEGO software was used to undertake GO functional classification for all the unigenes and to investigate the distribution of gene function in the species at the macro level [32]. The COG annotation was performed using the BLASTX algorithm (evalue threshold: 1e-5) against the Cluster of Orthologous Groups database. The KEGG pathways annotation was performed by sequence comparisons against the Kyoto Encyclopedia of Genes and Genomes database using BLASTX with an evalue threshold of 1e-5 [18].

### Normalization of genes expression levels and analysis of differential gene expression

The gene abundance estimation and DEGs analyssis were carried out by trinity toolkit [16], which required bowtie [33], RSEM [34] and edgeR [35]. Reads of each samples were aligned to the transcriptome assembly by bowtie with a maxium insert size of 800 (default). Then gene abundance was estimated by RSEM, using Fragments Per kb per Million fragments (FPKM) method [36]. The cut-off value for determining gene transcriptional activity was determined based on a 95% confidence interval for all FPKM values of each gene. An FPKM filtering cutoff of 1.0 in at least one of the four samples was used to determine expressed transcripts. DEGs were then analysised by R Bioconductor package edgeR and selected on condition of p-value ≤0.001 and |log_2_ (MS_RPKM/WS_RPKM)| ≥2. Hypergeometric test with Benjamini & Hochberg false discovery rate (FDR) were performed using the default parameters to adjust the P-value. GO category analysis was carried out using software Blast2GO mentioned above and GO functional enrichment analysis was carried out using GO-TermFinder [20]. KEGG pathway analyses of differentially expressed genes were performed using the KOBAS 2.0 (KEGG Orthology Based Annotation System) [21].

### Quantitative real-time PCR (qRT-PCR)

Real-time PCR reactions were set up with three biological replications and three technical replicates per experiment. The variance analysis (ANOVA) was performed for statistical analysis after logarithmic transformation of raw data. Total RNA was isolated from the samples and used for cDNA synthesis with the same procedures as detailed above. For qRT-PCR, the transcript levels of genes in the spathe of the wild type and the mutant were using the SYBR Green dye method. Each reaction buffer (10 μl) was composed of 50 ng of cDNA samples, 5 μl of 2× SYBR Green Master Mix Reagent (Applied Biosystems), and 0.2 μM of gene-specific primers (Table 4). Actin was used as an internal control to normalize the relative expression level of the analysed genes in wild type and the mutant anthurium, respectively. The thermal cycles used were as follows: 95 ℃ for 10 min, and 45 cycles of 95 ℃ for 5 s, 60 ℃ for 30 s. Each sample was amplified in four independent replicates. Relative gene expression was calculated according to the delta-delta Ct method of the system. The qPCR products were confirmed by both gel electrophoresis and sequencing.

**Table 4:** Primer sequences used for qRT-PCR analysis.

*Genes*	*Primers (5’-3’)*	
	Foward	Reverse
β-actin	ggtggagccacgacctta	ctctcattgggatggaagc
C50000092719_g1_i1	aacttgtggttcgtcttct	gaggttgaggcttgactata
c2000005858_g4_i1	ctacttcttgagattgtggatg	cctggtgatgctactactg
c5000006781_g1_i1	gccgaagaagaccagaag	gtagttgagccaccttagc
c2000001570_g2_i1	gtgtcagccataacattcg	catccaagccttccattct
c3000006903_g1_i1	gatgtaggcgtggttgtg	agtgagcttcttgatggatg
c2000004629_g2_i1	tggtgttcctctgtttcggg	atcgggttctggtggcttct
c30000038819_g1_i1	tccctcagagaccacaggaa	agacttccgcgccaagtt
c2000001891_g4_i1	aacaagttggctccttcc	acctcctccctctgaaag

### HPLC analysis of anthocyanin

The spathe, leaf and petiole of *A*. *andraeanum* (1 g for each tissue) were ground in 1.5 mL of 70% methanol containing 2% formic acid, then centrifuged at 14,000 rpm for 10 min at. Then the supernatant was filtered through a 0.45-μm syringe filter before HPLC analysis. Anthocyanins were investigated on an Shimadzu HPLC equipped with a SPD-6VA UV-detector.

### Plant Transformation and analysis of transgenic plants

The full-length cDNA for AN2 was cloned into the vector pMD18-T (Takara) under the control of the CaMV 35S promoter. The orientation of the plasmids was identified by PCR and used for further plant transformation. The plasmids were introduced in the Arabidopsis ecotype ‘Columbia’ plants using a floral dip method [37]. T1 seeds were screened on MS medium containing 50 mg/L Hyg. Then the positive seedlings were transferred to pots and grown in a growth chamber for futher analysis.

## Supporting Information

S1 Fig Figure of *35S*::*AnAN2* transgenic *A*. *thaliana* and wild-type *A*. *thaliana* seedling and adult plants. (A) wild-type *A*. *thaliana* seedling; (B) *35S*::*AnAN2* transgenic *A*. *thaliana* seedling; (C) *35S*::*AnAN2* transgenic *A*. *thaliana* adult plant (right) and wild-type *A*. *thaliana* adult plant (left).
(TIF)

S2 Fig Gel electrophoresis confirmation of qPCR products of *AN2*. M: marker.
(TIF)

S1 Table The summary of GO assignments of *A*. *andraeanum* transcriptome. (DOC)

S2 Table The summary of COG classfication of *A*. *andraeanum* transcriptome. (DOC)

S3 Table The summary of KEGG annotations of *A*. *andraeanum* transcriptome. (DOC)

S4 Table The summary of GO enrichment analysis of differently expressed genes. Bn (background number), indicates the total number of transcripts for certain pathways. Cp, corrected pvaluel use ‘bonferoni’ corretion. Nt1, the number of differently expressed genes in WS6 vs MS6. Nt2, the number of differently expressed genes in WS3 vs MS3. Nt3, the number of differently expressed genes in MS6 vs MS3. Nt4, the number of differently expressed genes in WS6 vs WS6.
(DOC)

S5 Table The summary of KEGG enrichment analysis of differently expressed genes. Bn (background number), indicates the total number of transcripts for certain pathways. Nt (number of transcripts), indicates the differently expressed genes for the certain pathways.
(DOC)

S6 Table Differentially expressed genes related with anthocyanin. (XLS)

S7 Table Peak value of three kinds of anthocyanidin in wild-type and mutant *A*. *andraeanum*. (DOC)
