# Supplementary figures and images for: Comparative Transcriptome Analysis of Anthurium “Albama” and Its Anthocyanin-Loss Mutant
Source: PLoS One. 2015 Mar 17;10(3):e0119027. doi: 10.1371/journal.pone.0119027 (PMC4363789; doi:10.1371/journal.pone.0119027)

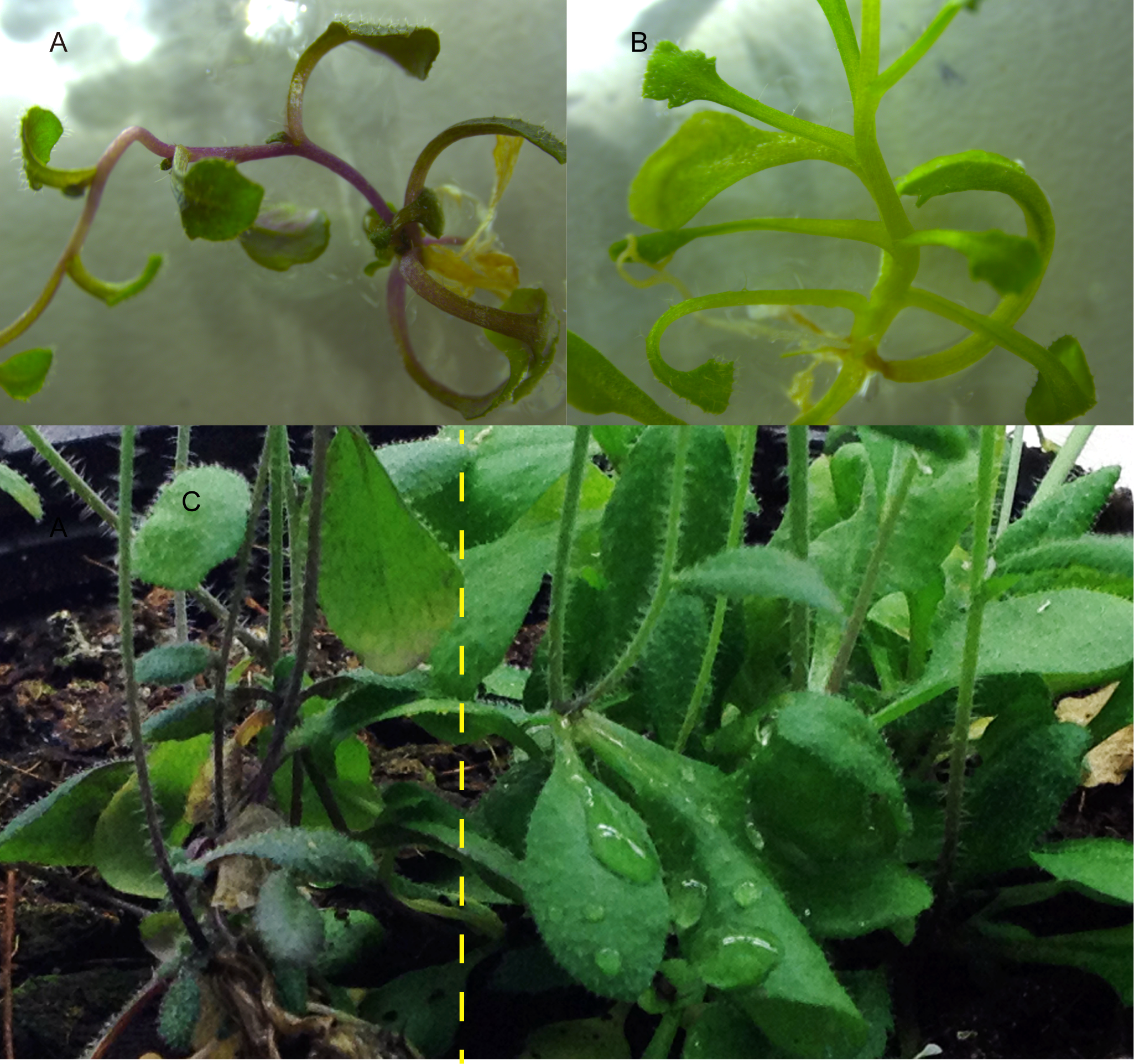

Supplement: S1 Fig — (A) wild-type A. thaliana seedling; (B) 35S::AnAN2 transgenic A. thaliana seedling; (C) 35S::AnAN2 transgenic A. thaliana adult plant (right) and wild-type A. thaliana adult plant (left). (TIF) [file pone.0119027.s001.tif]

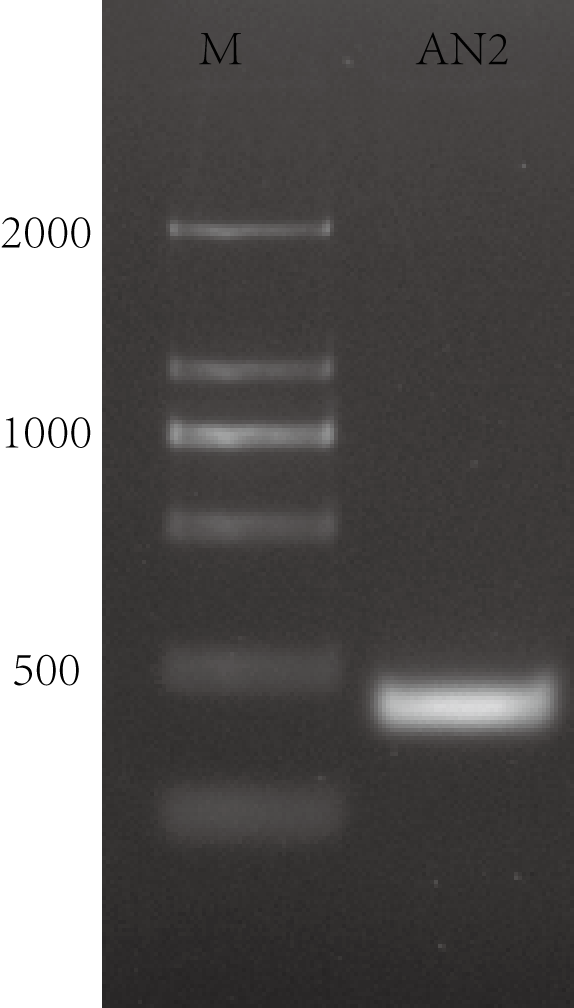

Supplement: S2 Fig — M: marker. (TIF) [file pone.0119027.s002.tif]
