# Supplementary material for: Comparative Transcriptome Analysis of Anthurium “Albama” and Its Anthocyanin-Loss Mutant
Source: PLoS One. 2015 Mar 17;10(3):e0119027. doi: 10.1371/journal.pone.0119027 (PMC4363789; doi:10.1371/journal.pone.0119027)
Supplement: S1 Table — (DOC) [file pone.0119027.s003.doc]

**S1 Table. The summary of GO assignments of anthurium transcriptome.**

| ***Ontology*** | ***Class*** | ***Number of Genes*** | ***Percent*** |
| --- | --- | --- | --- |
| molecular_function | antioxidant activity | 146 | 0.0749% |
| molecular_function | binding | 13656 | 7.0052% |
| molecular_function | catalytic activity | 13732 | 7.0442% |
| molecular_function | channel regulator activity | 1 | 0.0005% |
| molecular_function | electron carrier activity | 243 | 0.1247% |
| molecular_function | enzyme regulator activity | 245 | 0.1257% |
| molecular_function | metallochaperone activity | 5 | 0.0026% |
| molecular_function | molecular transducer activity | 420 | 0.2155% |
| molecular_function | nucleic acid binding transcription factor activity | 392 | 0.2011% |
| molecular_function | nutrient reservoir activity | 21 | 0.0108% |
| molecular_function | protein binding transcription factor activity | 41 | 0.0210% |
| molecular_function | protein tag | 1 | 0.0005% |
| molecular_function | receptor activity | 110 | 0.0564% |
| molecular_function | structural molecule activity | 639 | 0.3278% |
| molecular_function | translation regulator activity | 35 | 0.0180% |
| molecular_function | transporter activity | 1659 | 0.8510% |
| molecular_function | transcription regulator activity | 36 | 0.0185% |
| cellular_component | cell | 20693 | 10.6151% |
| cellular_component | cell junction | 645 | 0.3309% |
| cellular_component | cell part | 20693 | 10.6151% |
| cellular_component | extracellular matrix | 19 | 0.0097% |
| cellular_component | extracellular matrix part | 7 | 0.0036% |
| cellular_component | extracellular region | 1059 | 0.5432% |
| cellular_component | extracellular region part | 19 | 0.0097% |
| cellular_component | macromolecular complex | 2767 | 1.4194% |
| cellular_component | membrane | 6287 | 3.2251% |
| cellular_component | membrane part | 2177 | 1.1168% |
| cellular_component | membrane-enclosed lumen | 969 | 0.4971% |
| cellular_component | nucleoid | 65 | 0.0333% |
| cellular_component | organelle | 16782 | 8.6088% |
| cellular_component | organelle part | 4694 | 2.4079% |
| cellular_component | symplast | 645 | 0.3309% |
| cellular_component | virion | 9 | 0.0046% |
| cellular_component | virion part | 9 | 0.0046% |
| cellular_component | envelope | 202 | 0.1036% |
| biological_process | biological adhesion | 116 | 0.0595% |
| biological_process | biological regulation | 5040 | 2.5854% |
| biological_process | cellular component organization or biogenesis | 3579 | 1.8359% |
| biological_process | cellular process | 15412 | 7.9060% |
| biological_process | developmental process | 4083 | 2.0945% |
| biological_process | establishment of localization | 3598 | 1.8457% |
| biological_process | growth | 824 | 0.4227% |
| biological_process | immune system process | 706 | 0.3622% |
| biological_process | localization | 3737 | 1.9170% |
| biological_process | locomotion | 21 | 0.0108% |
| biological_process | metabolic process | 15167 | 7.7803% |
| biological_process | multi-organism process | 1595 | 0.8182% |
| biological_process | multicellular organismal process | 3908 | 2.0047% |
| biological_process | negative regulation of biological process | 1017 | 0.5217% |
| biological_process | positive regulation of biological process | 869 | 0.4458% |
| biological_process | regulation of biological process | 4144 | 2.1258% |
| biological_process | reproduction | 2433 | 1.2481% |
| biological_process | reproductive process | 2250 | 1.1542% |
| biological_process | response to stimulus | 6410 | 3.2882% |
| biological_process | rhythmic process | 114 | 0.0585% |
| biological_process | signaling | 1427 | 0.7320% |
| biological_process | single-organism process | 8373 | 4.2952% |
| biological_process | anatomical structure formation | 47 | 0.0241% |
| biological_process | cellular component biogenesis | 81 | 0.0416% |
| biological_process | cellular component organization | 383 | 0.1965% |
| biological_process | death | 39 | 0.0200% |
| biological_process | pigmentation | 443 | 0.2272% |
| biological_process | viral reproduction | 1 | 0.0005% |
