# Supplementary material for: Comparative Transcriptome Analysis of Anthurium “Albama” and Its Anthocyanin-Loss Mutant
Source: PLoS One. 2015 Mar 17;10(3):e0119027. doi: 10.1371/journal.pone.0119027 (PMC4363789; doi:10.1371/journal.pone.0119027)
Supplement: S2 Table — (DOC) [file pone.0119027.s004.doc]

**S2. Table The summary of COG classfication of anthurium transcriptome.**

| **Functional Code** | **Functional categories** | **Number of Genes** | **Percent (%)** |
| --- | --- | --- | --- |
| A | RNA processing and modification ; | 240 | 0.4264% |
| B | Chromatin structure and dynamics ; | 301 | 0.5347% |
| C | Energy production and conversion ; | 981 | 1.7428% |
| D | Cell cycle control, cell division, chromosome partitioning ; | 3906 | 6.9391% |
| E | Amino acid transport and metabolism ; | 1473 | 2.6168% |
| F | Nucleotide transport and metabolism ; | 510 | 0.9060% |
| G | Carbohydrate transport and metabolism ; | 2698 | 4.7930% |
| H | Coenzyme transport and metabolism ; | 553 | 0.9824% |
| I | Lipid transport and metabolism ; | 1254 | 2.2277% |
| J | Translation, ribosomal structure and biogenesis ; | 6359 | 11.2969% |
| K | Transcription ; | 5364 | 9.5292% |
| L | Replication, recombination and repair ; | 4732 | 8.4065% |
| M | Cell wall/membrane/envelope biogenesis ; | 3576 | 6.3528% |
| N | Cell motility ; | 522 | 0.9273% |
| O | Posttranslational modification, protein turnover, chaperones ; | 3476 | 6.1752% |
| P | Inorganic ion transport and metabolism ; | 963 | 1.7108% |
| Q | Secondary metabolites biosynthesis, transport and catabolism ; | 1434 | 2.5475% |
| R | General function prediction only ; | 6586 | 11.7001% |
| S | Function unknown ; | 4665 | 8.2874% |
| T | Signal transduction mechanisms ; | 3413 | 6.0632% |
| U | Intracellular trafficking, secretion, and vesicular transport ; | 2143 | 3.8071% |
| V | Defense mechanisms ; | 13 | 0.0231% |
| W | Extracellular structures | 517 | 0.9185% |
| Y | Nuclear structure | 2 | 0.0036% |
| Z | Cytoskeleton ; | 609 | 1.0819% |
