# Supplementary material for: Comparative Transcriptome Analysis of Anthurium “Albama” and Its Anthocyanin-Loss Mutant
Source: PLoS One. 2015 Mar 17;10(3):e0119027. doi: 10.1371/journal.pone.0119027 (PMC4363789; doi:10.1371/journal.pone.0119027)
Supplement: S3 Table — (DOC) [file pone.0119027.s005.doc]

**S3 Table. The summary of KEGG annotations of anthurium** transcriptome.

| **Pathway** | **Pathway.ID** | **Number of Genes** | **Percent** |
| --- | --- | --- | --- |
| Metabolic pathways | ko01100 | 7258 | 16.5432% |
| RNA transport | ko03013 | 1910 | 4.3535% |
| Endocytosis | ko04144 | 1967 | 4.4834% |
| Glycerophospholipid metabolism | ko00564 | 1891 | 4.3102% |
| Biosynthesis of secondary metabolites | ko01110 | 2381 | 5.4270% |
| Spliceosome | ko03040 | 3424 | 7.8043% |
| Ether lipid metabolism | ko00565 | 1737 | 3.9592% |
| mRNA surveillance pathway | ko03015 | 1442 | 3.2868% |
| Plant-pathogen interaction | ko04626 | 1595 | 3.6355% |
| Plant hormone signal transduction | ko04075 | 798 | 1.8189% |
| Purine metabolism | ko00230 | 1113 | 2.5369% |
| Starch and sucrose metabolism | ko00500 | 856 | 1.9511% |
| Pyrimidine metabolism | ko00240 | 1065 | 2.4275% |
| Ribosome biogenesis in eukaryotes | ko03008 | 650 | 1.4815% |
| Protein processing in endoplasmic reticulum | ko04141 | 669 | 1.5249% |
| RNA degradation | ko03018 | 650 | 1.4815% |
| RNA polymerase | ko03020 | 828 | 1.8873% |
| Pentose and glucuronate interconversions | ko00040 | 534 | 1.2171% |
| Ribosome | ko03010 | 550 | 1.2536% |
| Ubiquitin mediated proteolysis | ko04120 | 453 | 1.0325% |
| Glycolysis / Gluconeogenesis | ko00010 | 330 | 0.7522% |
| Oxidative phosphorylation | ko00190 | 315 | 0.7180% |
| Amino sugar and nucleotide sugar metabolism | ko00520 | 302 | 0.6884% |
| ABC transporters | ko02010 | 232 | 0.5288% |
| Phagosome | ko04145 | 275 | 0.6268% |
| Phenylpropanoid biosynthesis | ko00940 | 299 | 0.6815% |
| Galactose metabolism | ko00052 | 202 | 0.4604% |
| Pyruvate metabolism | ko00620 | 262 | 0.5972% |
| Homologous recombination | ko03440 | 220 | 0.5014% |
| Basal transcription factors | ko03022 | 206 | 0.4695% |
| Peroxisome | ko04146 | 242 | 0.5516% |
| Nucleotide excision repair | ko03420 | 237 | 0.5402% |
| Circadian rhythm - plant | ko04712 | 215 | 0.4901% |
| Phosphatidylinositol signaling system | ko04070 | 187 | 0.4262% |
| Glycine, serine and threonine metabolism | ko00260 | 156 | 0.3556% |
| Fructose and mannose metabolism | ko00051 | 169 | 0.3852% |
| Pentose phosphate pathway | ko00030 | 160 | 0.3647% |
| Cysteine and methionine metabolism | ko00270 | 443 | 1.0097% |
| Inositol phosphate metabolism | ko00562 | 162 | 0.3692% |
| Other glycan degradation | ko00511 | 137 | 0.3123% |
| Aminoacyl-tRNA biosynthesis | ko00970 | 155 | 0.3533% |
| Glyoxylate and dicarboxylate metabolism | ko00630 | 129 | 0.2940% |
| Cyanoamino acid metabolism | ko00460 | 157 | 0.3579% |
| Carotenoid biosynthesis | ko00906 | 158 | 0.3601% |
| Flavonoid biosynthesis | ko00941 | 189 | 0.4308% |
| Sphingolipid metabolism | ko00600 | 129 | 0.2940% |
| Carbon fixation in photosynthetic organisms | ko00710 | 160 | 0.3647% |
| Glutathione metabolism | ko00480 | 143 | 0.3259% |
| Regulation of autophagy | ko04140 | 126 | 0.2872% |
| DNA replication | ko03030 | 162 | 0.3692% |
| Mismatch repair | ko03430 | 166 | 0.3784% |
| Glycerolipid metabolism | ko00561 | 149 | 0.3396% |
| Fatty acid metabolism | ko00071 | 154 | 0.3510% |
| Arginine and proline metabolism | ko00330 | 150 | 0.3419% |
| Porphyrin and chlorophyll metabolism | ko00860 | 145 | 0.3305% |
| Terpenoid backbone biosynthesis | ko00900 | 125 | 0.2849% |
| Base excision repair | ko03410 | 146 | 0.3328% |
| N-Glycan biosynthesis | ko00510 | 127 | 0.2895% |
| Protein export | ko03060 | 108 | 0.2462% |
| Phenylalanine metabolism | ko00360 | 141 | 0.3214% |
| Stilbenoid, diarylheptanoid and gingerol biosynthesis | ko00945 | 176 | 0.4012% |
| Valine, leucine and isoleucine degradation | ko00280 | 133 | 0.3031% |
| Limonene and pinene degradation | ko00903 | 182 | 0.4148% |
| Ascorbate and aldarate metabolism | ko00053 | 125 | 0.2849% |
| Nitrogen metabolism | ko00910 | 114 | 0.2598% |
| Tyrosine metabolism | ko00350 | 114 | 0.2598% |
| Zeatin biosynthesis | ko00908 | 125 | 0.2849% |
| Propanoate metabolism | ko00640 | 134 | 0.3054% |
| Proteasome | ko03050 | 108 | 0.2462% |
| alpha-Linolenic acid metabolism | ko00592 | 109 | 0.2484% |
| Phenylalanine, tyrosine and tryptophan biosynthesis | ko00400 | 95 | 0.2165% |
| Ubiquinone and other terpenoid-quinone biosynthesis | ko00130 | 103 | 0.2348% |
| Valine, leucine and isoleucine biosynthesis | ko00290 | 107 | 0.2439% |
| Citrate cycle (TCA cycle) | ko00020 | 119 | 0.2712% |
| Glycosylphosphatidylinositol(GPI)-anchor biosynthesis | ko00563 | 76 | 0.1732% |
| Photosynthesis | ko00195 | 110 | 0.2507% |
| Steroid biosynthesis | ko00100 | 85 | 0.1937% |
| Fatty acid biosynthesis | ko00061 | 119 | 0.2712% |
| One carbon pool by folate | ko00670 | 71 | 0.1618% |
| SNARE interactions in vesicular transport | ko04130 | 83 | 0.1892% |
| Glycosaminoglycan degradation | ko00531 | 76 | 0.1732% |
| Alanine, aspartate and glutamate metabolism | ko00250 | 94 | 0.2143% |
| Flavone and flavonol biosynthesis | ko00944 | 84 | 0.1915% |
| Cutin, suberine and wax biosynthesis | ko00073 | 60 | 0.1368% |
| Pantothenate and CoA biosynthesis | ko00770 | 77 | 0.1755% |
| Sulfur metabolism | ko00920 | 78 | 0.1778% |
| Biosynthesis of unsaturated fatty acids | ko01040 | 96 | 0.2188% |
| Fatty acid elongation | ko00062 | 60 | 0.1368% |
| Lysine degradation | ko00310 | 96 | 0.2188% |
| beta-Alanine metabolism | ko00410 | 77 | 0.1755% |
| Natural killer cell mediated cytotoxicity | ko04650 | 65 | 0.1482% |
| Tryptophan metabolism | ko00380 | 92 | 0.2097% |
| Circadian rhythm - mammal | ko04710 | 43 | 0.0980% |
| Butanoate metabolism | ko00650 | 76 | 0.1732% |
| Diterpenoid biosynthesis | ko00904 | 51 | 0.1162% |
| Glycosphingolipid biosynthesis - ganglio series | ko00604 | 46 | 0.1048% |
| Linoleic acid metabolism | ko00591 | 69 | 0.1573% |
| Lysine biosynthesis | ko00300 | 43 | 0.0980% |
| Histidine metabolism | ko00340 | 65 | 0.1482% |
| Tropane, piperidine and pyridine alkaloid biosynthesis | ko00960 | 42 | 0.0957% |
| Glucosinolate biosynthesis | ko00966 | 52 | 0.1185% |
| Isoquinoline alkaloid biosynthesis | ko00950 | 38 | 0.0866% |
| Non-homologous end-joining | ko03450 | 40 | 0.0912% |
| Selenocompound metabolism | ko00450 | 64 | 0.1459% |
| Folate biosynthesis | ko00790 | 42 | 0.0957% |
| Brassinosteroid biosynthesis | ko00905 | 38 | 0.0866% |
| Photosynthesis - antenna proteins | ko00196 | 41 | 0.0935% |
| Arachidonic acid metabolism | ko00590 | 28 | 0.0638% |
| Nicotinate and nicotinamide metabolism | ko00760 | 39 | 0.0889% |
| Riboflavin metabolism | ko00740 | 38 | 0.0866% |
| Benzoxazinoid biosynthesis | ko00402 | 42 | 0.0957% |
| Other types of O-glycan biosynthesis | ko00514 | 20 | 0.0456% |
| Sulfur relay system | ko04122 | 26 | 0.0593% |
| Glycosphingolipid biosynthesis - globo series | ko00603 | 27 | 0.0615% |
| Taurine and hypotaurine metabolism | ko00430 | 19 | 0.0433% |
| Monoterpenoid biosynthesis | ko00902 | 28 | 0.0638% |
| Sesquiterpenoid and triterpenoid biosynthesis | ko00909 | 16 | 0.0365% |
| Vitamin B6 metabolism | ko00750 | 16 | 0.0365% |
| Isoflavonoid biosynthesis | ko00943 | 14 | 0.0319% |
| Indole alkaloid biosynthesis | ko00901 | 26 | 0.0593% |
| Thiamine metabolism | ko00730 | 17 | 0.0387% |
| Synthesis and degradation of ketone bodies | ko00072 | 16 | 0.0365% |
| Biotin metabolism | ko00780 | 17 | 0.0387% |
| Lipoic acid metabolism | ko00785 | 13 | 0.0296% |
| Caffeine metabolism | ko00232 | 9 | 0.0205% |
| Anthocyanin biosynthesis | ko00942 | 11 | 0.0251% |
| C5-Branched dibasic acid metabolism | ko00660 | 10 | 0.0228% |
| Betalain biosynthesis | ko00965 | 5 | 0.0114% |
| Polyketide sugar unit biosynthesis | ko00523 | 2 | 0.0046% |
