# Supplementary material for: Comparative Transcriptome Analysis of Anthurium “Albama” and Its Anthocyanin-Loss Mutant
Source: PLoS One. 2015 Mar 17;10(3):e0119027. doi: 10.1371/journal.pone.0119027 (PMC4363789; doi:10.1371/journal.pone.0119027)
Supplement: S4 Table — Bn (background number), indicates the total number of transcripts for certain pathways. Cp, corrected pvaluel use ‘bonferoni’ corretion. Nt1, the number of differently expressed genes in WS6 vs MS6. Nt2, the number of differently expressed genes in WS3 vs MS3. Nt3, the number of differently expressed genes in MS6 vs MS3. Nt4, the number of differently expressed genes in WS6 vs WS6. (DOC) [file pone.0119027.s006.doc]

**S4 Table. The summary of GO enrichment analysis of differently expressed genes.**

| ***Ontology*** | ***Class*** | ***Bn*** | ***Nt1*** | ***Cp*** | ***Nt2*** | ***Cp*** | ***N3t*** | ***Cp*** | ***N4t*** | ***Cp*** |
| --- | --- | --- | --- | --- | --- | --- | --- | --- | --- | --- |
| molecular_function | catalytic activity | 13986 | 148 |  | 276 | 0.001115 | 934 |  | 1003 |  |
| molecular_function | nucleic acid binding transcription factor activity | 399 | 1 |  | 3 |  | 44 | 0.024683 | 47 | 0.036096 |
| molecular_function | binding | 13910 | 133 |  | 219 |  | 857 | 0.00072 | 982 |  |
| cellular_component | virion | 9 | 2 | 0.006264 | 0 |  | 2 |  | 2 |  |
| cellular_component | virion part | 9 | 2 | 0.006264 | 0 |  | 2 |  | 2 |  |
| cellular_component | membrane | 6439 | 61 |  | 137 | 0.019912 | 488 |  | 534 |  |
| cellular_component | cell junction | 658 | 8 |  | 15 |  | 69 | 0.008035 | 72 | 0.029628 |
| cellular_component | nucleoid | 66 | 2 |  | 0 |  | 1 | 0.044784 | 6 |  |
| cellular_component | symplast | 658 | 8 |  | 15 |  | 69 | 0.008035 | 72 | 0.029628 |
| cellular_component | cell | 21055 | 210 |  | 341 |  | 1372 | 0.036551 | 1564 |  |
| cellular_component | cell part | 21055 | 210 |  | 341 |  | 1372 | 0.036551 | 1564 |  |
| cellular_component | extracellular region | 1083 | 9 |  | 16 |  | 141 | 7.39E-12 | 135 | 4.15E-07 |
| cellular_component | organelle | 17076 | 171 |  | 288 |  | 1083 | 0.004715 | 1247 |  |
| cellular_component | envelope | 203 | 1 |  | 1 |  | 3 | 0.000883 | 5 | 0.005479 |
| biological_process | metabolic process | 15464 | 171 |  | 293 | 0.015733 | 1107 |  | 1256 | 0.025034 |
| biological_process | cellular component organization | 383 | 3 |  | 1 | 0.010859 | 3 | 1.51E-08 | 8 | 1.18E-05 |
| biological_process | cellular component organization or biogenesis | 3651 | 38 |  | 67 |  | 379 | 1.33E-15 | 421 | 1.08E-17 |
| biological_process | negative regulation of biological process | 1032 | 10 |  | 20 |  | 125 | 1.92E-08 | 144 | 4.70E-11 |
| biological_process | regulation of biological process | 4230 | 54 |  | 70 |  | 432 | 6.46E-17 | 473 | 1.28E-17 |
| biological_process | single-organism process | 8565 | 88 |  | 168 |  | 828 | 1.22E-30 | 905 | 3.61E-31 |
| biological_process | biological regulation | 5129 | 63 |  | 83 |  | 476 | 2.31E-11 | 522 | 9.77E-12 |
| biological_process | cellular process | 15717 | 162 |  | 272 |  | 1159 | 0.01157 | 1313 | 1.11E-05 |
| biological_process | developmental process | 4165 | 39 |  | 80 |  | 407 | 1.22E-12 | 434 | 7.37E-11 |
| biological_process | growth | 845 | 9 |  | 15 |  | 85 | 0.007807 | 90 | 0.021715 |
| biological_process | multicellular organismal process | 3985 | 38 |  | 75 |  | 385 | 4.02E-11 | 416 | 1.94E-10 |
| biological_process | reproduction | 2485 | 21 |  | 33 |  | 243 | 4.80E-07 | 268 | 1.25E-07 |
| biological_process | reproductive process | 2301 | 21 |  | 33 |  | 232 | 7.81E-08 | 255 | 2.50E-08 |
| biological_process | response to stimulus | 6546 | 58 |  | 125 |  | 514 | 0.016587 | 570 | 0.007535 |
| biological_process | pigmentation | 445 | 4 |  | 5 |  | 14 | 0.003813 | 18 | 0.01413 |
| biological_process | cellular component biogenesis | 81 | 2 |  | 0 |  | 2 |  | 1 | 0.011062 |

Bn (background number), indicates the total number of transcripts for certain pathways. Cp, corrected pvaluel use ‘bonferoni’ corretion. Nt1, the number of differently expressed genes in WS6 vs MS6. Nt2, the number of differently expressed genes in WS3 vs MS3. Nt3, the number of differently expressed genes in MS6 vs MS3. Nt4, the number of differently expressed genes in WS6 vs WS3.
