# Supplementary material for: Comparative Transcriptome Analysis of Anthurium “Albama” and Its Anthocyanin-Loss Mutant
Source: PLoS One. 2015 Mar 17;10(3):e0119027. doi: 10.1371/journal.pone.0119027 (PMC4363789; doi:10.1371/journal.pone.0119027)
Supplement: S5 Table — Bn (background number), indicates the total number of transcripts for certain pathways. Nt (number of transcripts), indicates the differently expressed genes for the certain pathways. (DOC) [file pone.0119027.s007.doc]

**S5 Table. The summary of KEGG enrichment analysis of differently expressed genes.**

| ***Pathway*** | ***Pathway.ID*** | ***bn*** | ***nt*** | ***Cp*** | ***nt*** | ***cp*** | ***Nt*** | ***Cp*** | ***nt*** | ***cp*** |
| --- | --- | --- | --- | --- | --- | --- | --- | --- | --- | --- |
| Purine metabolism | ko00230 | 448 | 10 | 0.049014 | 3 |  | 42 |  | 33 |  |
| RNA polymerase | ko03020 | 555 | 1 | 0.033354 | 4 |  | 20 | 0.001269 | 27 | 0.036247 |
| Homologous recombination | ko03440 | 1927 | 8 | 0.042573 | 14 | 0.001656 | 256 | 1.10E-18 | 239 | 7.40E-11 |
| Phosphatidylinositol signaling system | ko04070 | 539 | 1 | 0.037478 | 4 |  | 58 |  | 64 | 0.017706 |
| Glyoxylate and dicarboxylate metabolism | ko00630 | 214 | 9 | 0.001046 | 5 |  | 12 |  | 10 |  |
| Thiamine metabolism | ko00730 | 12 | 2 | 0.009605 | 1 |  | 0 |  | 1 |  |
| Ribosome biogenesis in eukaryotes | ko03008 | 663 | 7 |  | 3 | 0.010109 | 20 | 1.03E-05 | 29 | 0.002519 |
| Cutin, suberine and wax biosynthesis | ko00073 | 63 | 1 |  | 7 | 0.000503 | 22 | 6.38E-09 | 9 |  |
| Endocytosis | ko04144 | 3461 | 23 |  | 32 | 0.001282 | 118 | 4.34E-24 | 156 | 2.02E-15 |
| Biosynthesis of secondary metabolites | ko01110 | 1624 | 12 |  | 8 | 6.94E-05 | 96 |  | 118 |  |
| Aminoacyl-tRNA biosynthesis | ko00970 | 240 | 5 |  | 10 | 0.0467 | 21 |  | 22 |  |
| Ubiquinone and other terpenoid-quinone biosynthesis | ko00130 | 1771 | 8 |  | 14 | 0.007539 | 250 | 8.24E-22 | 232 | 5.21E-13 |
| RNA transport | ko03013 | 1955 | 11 |  | 25 |  | 211 | 1.11E-06 | 232 | 1.14E-08 |
| mRNA surveillance pathway | ko03015 | 1467 | 6 |  | 19 |  | 178 | 2.68E-09 | 183 | 2.20E-08 |
| Plant hormone signal transduction | ko04075 | 819 | 12 |  | 20 |  | 112 | 1.25E-08 | 106 | 1.43E-05 |
| Metabolic pathways | ko01100 | 7383 | 72 |  | 112 |  | 729 | 1.71E-17 | 690 | 1.11E-05 |
| Glycerophospholipid metabolism | ko00564 | 2426 | 29 |  | 53 |  | 231 | 0.002342 | 235 | 0.026396 |
| Starch and sucrose metabolism | ko00500 | 866 | 5 |  | 11 |  | 94 | 0.004545 | 94 | 0.031361 |
| Protein processing in endoplasmic reticulum | ko04141 | 660 | 6 |  | 6 |  | 30 | 0.021659 | 35 |  |
| Pentose and glucuronate interconversions | ko00040 | 2009 | 9 |  | 20 |  | 255 | 5.00E-16 | 251 | 8.57E-12 |
| Oxidative phosphorylation | ko00190 | 304 | 2 |  | 8 |  | 51 | 1.09E-06 | 45 | 0.00079 |
| Peroxisome | ko04146 | 191 | 2 |  | 4 |  | 5 | 0.013189 | 9 |  |
| Fructose and mannose metabolism | ko00051 | 121 | 2 |  | 1 |  | 3 | 0.039342 | 1 | 0.000489 |
| Other glycan degradation | ko00511 | 192 | 3 |  | 5 |  | 29 | 0.003332 | 30 | 0.004204 |
| Flavonoid biosynthesis | ko00941 | 162 | 1 |  | 0 |  | 36 | 5.61E-08 | 36 | 2.79E-07 |
| Regulation of autophagy | ko04140 | 120 | 1 |  | 0 |  | 3 | 0.041449 | 2 | 0.005389 |
| DNA replication | ko03030 | 144 | 1 |  | 3 |  | 27 | 0.000124 | 20 |  |
| Phenylalanine metabolism | ko00360 | 161 | 3 |  | 7 |  | 22 | 0.046974 | 22 |  |
| Nitrogen metabolism | ko00910 | 97 | 1 |  | 0 |  | 1 | 0.004371 | 6 |  |
| Fatty acid biosynthesis | ko00061 | 77 | 1 |  | 1 |  | 1 | 0.016265 | 3 |  |
| SNARE interactions in vesicular transport | ko04130 | 159 | 3 |  | 5 |  | 30 | 3.80E-05 | 25 | 0.010169 |
| Sulfur metabolism | ko00920 | 132 | 0 |  | 1 |  | 3 | 0.021873 | 6 |  |
| Biosynthesis of unsaturated fatty acids | ko01040 | 138 | 4 |  | 1 |  | 25 | 0.000446 | 20 |  |
| ABC transporters | ko02010 | 245 | 4 |  | 7 |  | 12 |  | 9 | 0.029671 |
| Glutathione metabolism | ko00480 | 149 | 2 |  | 1 |  | 18 |  | 22 | 0.038659 |
| Propanoate metabolism | ko00640 | 95 | 0 |  | 1 |  | 4 |  | 2 | 0.026463 |

Bn (background number), indicates the total number of transcripts for certain pathways. Nt (number of transcripts), indicates the number differently expressed genes for the certain pathways.
