# Supplementary material for: Comparative Transcriptome Analysis of Anthurium “Albama” and Its Anthocyanin-Loss Mutant
Source: PLoS One. 2015 Mar 17;10(3):e0119027. doi: 10.1371/journal.pone.0119027 (PMC4363789; doi:10.1371/journal.pone.0119027)
Supplement: S7 Table — (DOC) [file pone.0119027.s009.doc]

**S9 Table. Peak value of three kinds of anthocyanidin in wild-type and mutant *A. andraeanum***.

| ***Tissue*** | ***Peonidin- rutinoside*** | ***Anthocyanidin- rutinoside*** | Pelargonidin- rutinoside |
| --- | --- | --- | --- |
| Wild-type spathe | 8039.45 | 1681.39 | 472.83 |
| Mutant spathe | - | - | - |
| Wild-type petiole | 135.59 | 26.64 | 10.77 |
| Mutant petiole | - | - | - |
| Wild-type leaf | 80.52 | 22.93 | 3.86 |
| Mutant leaf | - | - | - |
